# Influence of at-risk family interactions on the course of psychiatric care in adolescence

**DOI:** 10.1007/s00787-023-02330-5

**Published:** 2024-02-02

**Authors:** M. Robin, L. Surjous, J. Belbèze, L. Bonnardel, M. Varlet, J. Silva, J. Lamothe, A. Essadek, B. Falissard, D. Cohen, M. Corcos

**Affiliations:** 1 Department of Adolescent and Young Adult Psychiatry, Institut Mutualiste Montsouris, 42 Boulevard Jourdan, 75014 Paris, France; 2 Paris-Saclay University, UVSQ, INSERM U1178, Team PsyDev, Villejuif, France; 3 Paris Descartes University, Sorbonne Paris Cité, Paris, France; 4 Group for Research and Intervention on Children’s Social Adjustment (GRISE), Sherbrooke, Québec Canada; 5 Interpsy Laboratory, University of Lorraine, Nancy, France; 6 AP-HP, Hôpital Cochin, Maison de Solenn, Paris, France; 7 Department of Child and Adolescent Psychiatry, Sorbonne Université, Hôpital Pitié-Salpêtrière, Paris, France

**Keywords:** Family interactions, Adolescence, Psychiatric care, Hospitalization, ARFIL scale

## Abstract

Dysparenting, referring to inappropriate parental attitudes, is a vulnerability factor for mental disorders during adolescence and a therapeutic leverage, yet clinicians lack reliable tools to assess it in daily clinical practice. Moreover, the effect of this dysparenting on the amount of psychiatric care remains unclear. The Family and Care study aims to develop the at-risk family interactions and levers (ARFIL) scale, a comprehensive 30-item clinical scale, and to assess in a cross-sectional design, the impact of these at-risk family interactions on the care of adolescents (*n* = 425) hospitalized in psychiatry and aged 13–19 years old. Factorial analysis shows that the ARFIL scale consists of three main dimensions associated with cohesion/conflicts, love/hostility, and autonomy/control with good psychometric properties. Multivariate regressions show that the ARFIL intensity score predicts the duration of hospital care, regardless of age, gender, medical severity on admission, assessed by the Global Assessment of Functioning scale, the presence of maltreatment and psychiatric diagnoses. Moreover, the ARFIL diversity score (number of items present regardless of their severity) predicts both the number and duration of hospitalizations. At-risk family interactions are a determining dimension of psychiatric adolescent care, and the ARFIL scale could constitute a valuable tool, not only for holistic evaluation and treatment, but also for prevention.

**Supplementary Information:**

The online version contains supplementary material available at 10.1007/s00787-023-02330-5.

## Introduction

Child mental health is now recognized as a key public health concern, as it is estimated that 22–40% of adolescents aged 13–18 suffer from a mental disorder [1]. Alongside genetic and biological risk factors, the role of family in the prevention of these disorders, their severity, and in the course of their treatment has been described in a number of studies [2–7]. Some of them focus on the severe and long-lasting effects of maltreatment (abuse and neglect), on mental health [8], others focus on parenting styles. Among these, some dimensions are regularly described, like control or autonomy granting, over-involvement, inconsistency, warmth (affection, attentiveness, acceptance), family cohesion or communication level. Historically, two fundamental dimensions of parenting have been proposed by Schaefer [9]: love–hostility and control–autonomy. The first dimension refers to the parent’s ability to be accepting, supportive, and sensitive to the child’s needs. The second dimension refers to parental efforts to monitor and adjust their child’s behavior. According to Baumrind [10], it allows distinguishing four categories of parenting: authoritative (focus on limit setting with connection), authoritarian (focus on discipline without connection), permissive (lot of connections, low boundaries), and uninvolved (low connection and boundaries). In depressive and anxiety disorders, more inter-parental conflict, over-involvement, aversiveness, and less warmth have been described; and for depression, it also included less autonomy granting [4]. According to Pinquart, externalized and internalized disorders were predicted by harsh control, psychological control, authoritarian, and neglectful parenting (added to permissive parenting for externalized disorders) [6, 7]. Most scales focus on general parenting styles, but as studies delve deeper into the links between parenting and child psychiatric psychopathology, the need arises for more precise criteria of family interactions. Thus, significant unmeasured dimensions were revealed by other studies, such as inter-parental conflicts, premature separations or parental suicidal threats, particularly in their links with borderline pathology [11, 12].

Among the psychometric tools existing to measure parenting or family functioning according to these dimensions or these categories, most are self-administered questionnaires to be completed by the child, as the Parental Bonding Instrument (PBI) scale or the Parental Authority Questionnaire [13, 14]. Some others are to be completed by the parents as the Parental Self-Efficacy for Reducing the Risk of Adolescent Depression and Anxiety scale, the family Adaptability and Cohesion Evaluation Scale, or the Multidimensional Assessment of Parenting Scale [15–17]. But according to studies and experts in the field, the correlation between self-measured and child-reported parenting is weak. Most of the time, parents are more likely to identify difficulties arising from their children or from symptoms, and children are more likely to rate difficulties arising from their parents [18, 19]. Parents may be, for example, less aware of or inclined to report neglecting behavior or the use of psychological control strategies [16]. Measurement tools of family interactions observed by an outside observer have also been developed, as the Parenting Clinical Observation Schedule and the Iowa Family Interactions Rating Scale [20, 21]. The first one assesses three dimensions: responsive involvement, constructive discipline, and problematic discipline, in the context of a specific clinical profile (child disruptive behaviors). The second is focused on communication in parent dyad’s relationship and problem solving. These types of observational measurements of parenting usually demonstrate stronger prediction of child outcomes and better reliability than self-questionnaires [22, 23]. However, these scales use complex coding system (not suitable for clinical daily use), and do not capture parenting in a way that is sufficiently useful to everyday practice [20]. This lack of feasibility and relevance in clinical practice also highlights the limits of separated research from clinical practice in this area and calls for the development of tools that combine these different needs.

Up to now, studies have described general aspects of parental or family functioning in terms of communication, cohesion, and educative aspects. But on one hand, those parenting scales measure parent–child interactions but not parent–parent interactions (e.g., parenting conflicts regarding the child), and on the other hand, day-to-day clinical practice requires a more precise assessment of relational issues to better target therapy. For example, emotional invalidation (described in cognitive behavioral theory) or paradoxical communications (double-bind described in systemic theory) are very specific relational modalities, which can disorganize the child development if they are not accurately identified and dealt with. Considering the important role played by the family in the risk and resilience factors for mental disorders in children and adolescents, we can assume that a reliable measure of dysfunctional interactions could also be an important determinant, or even indicator, of the amount of care that will be needed for a given patient. To date, research into the predictive factors for psychiatric care and hospitalization in adolescent psychiatry has focused mainly on the clinical characteristics of the patient [24–26], family composition and history [27, 28], or abuse and neglect [26, 29, 30]. The few studies that have measured the specific links between parenting and psychiatric care described that family cohesion and support (through one or two dimensions) reduced the risk of hospitalization [31, 32]. Rice and Tan [33] described four main family factors precipitating the crisis leading to adolescent hospitalization: changes in family structure, exposure to family trauma, family conflict. and parental instability. But to our knowledge, no study has investigated the influence of the quality of parenthood on the amount of care a patient requires, while it is considered as an important healthy, politic, and economic challenge [34].

Considering both the importance of precisely assessing familial relationships in child and adolescents with mental health problems and the limitations of existing tools, we have created the at-risk family interactions and levers (ARFIL) scale. It is a hetero-evaluation by the clinician of the patient’s family relational dysfunctions, whose dimensions stem from previous scales, from theoretical background, and from clinical experience of a variety of psychiatric disorders in adolescence (Annex 1). The ARFIL scale makes it possible to base the assessment both on the emotions, behaviors, and comments of the youth and the parents, but also on the observation of clinicians. Moreover, this scale meets a current need to increasingly consider patients in their environment, to precisely analyze their needs and enable better-targeted, and therefore more effective, preventive, and therapeutic actions to be put in place. It is part of the Global health movement, which emphasizes transnational health issues, determinants, and solutions to improve health, through a synthesis of population-based prevention with individual-level clinical care [35].

The aims of this work are to present the psychometric criteria of the at-risk family interactions scale, and to test the hypothesis of the Family and Care study, that these interactions would increase the number and duration of adolescents’ psychiatric hospitalizations.

## Methods

### Participants

The Family and Care study is a cross-sectional study exploring the family dynamics of all patients hospitalized in the psychiatric care unit for four successive years, i.e., *n* = 425 patients aged 13–19 years. Patients come from center/south of Paris and the surrounding region, which has a higher standard of living than the regional average. Patients are referred for all types of psychiatric disorders by their psychiatrists or by local hospital emergency departments. Admissions are scheduled 48 h in advance on average. There was no change in the modalities of care or observation with a usual situation. At the end of care, the clinician rated the ARFIL scale and recorded other information in the patient record. The patients were seen every other day for a medical interview and for a family interview on admission, every week and on discharge, i.e., four 1-h family interviews on average. The ARFIL scale was coded by each of the two physicians on the unit for the patients for whom he was responsible, at discharge. The two doctors in the hospitalization unit checked the consistency of their respective ratings once a month, as collective teamwork made it possible to know all the patients (very regular communication meetings between colleagues, and meetings with patients). Alongside the ARFIL scale, the sociodemographic characteristics (age, sex, socioprofessional category) of the patients were noted, as well as the maltreatment reported by the patients, the parents, and/or identified by the nursing staff (emotional, physical and sexual abuse as well as neglect), according to the European CAN hetero-evaluation scale via MDS [36]. Socioeconomic status (SES) was determined according to each parent’s employment status. A dimensional index was developed as follows: (1) higher managerial/administrative/professional, (2) intermediate occupation, (3) unemployed and retired. The inclusion criterion was to be hospitalized in the department during the period studied. The only exclusion criterion was premature discharge that did not allow sufficient family assessment, i.e., discharge before 10 days of hospitalization (*n* = 7 patients). For disorders diagnoses according to the DSM 5 [37], patients completed the French version of the Mini-International Neuropsychiatric Interview (MINI) and of the Structured Interview for DSM Personality disorders for the nine criteria of BPD [38, 39]. Parents and adolescents both provided their written informed consent and procedures were approved by the Ethics Committee CPP-Ile de France II, Paris, France (n°20130910). The consent form indicated that adolescents and families were taking part in research into psychic vulnerability factors. Ethical conditions were met by providing totally routine care and immediately anonymizing the information collected by a research file number assigned to each patient. The scale was scored on paper and then entered anonymously into a computer (Excel), along with other data from the patient’s care file.

### Characteristics and construction of the scale

The ARFIL scale is a hetero-evaluation tool of 30 items describing different aspects of family functioning, that were generated from dimensions highlighted in a comprehensive way in child and adolescent psychiatry: education theories, attachment theory, cognitive and behavioral, psychoanalysis, systemic and transgenerational theory. It integrates parenting and familial dimensions described in previous studies. The ARFIL scale includes the affective and behavioral dimensions constitutive of the four parenting styles of the PBI, the Parental Authority Questionnaire and the Multidimensional Assessment of Parenting Scale derived from Schaefer and Baumrind [9, 10], such as inconsistent discipline (items 1, 4, 21), permissive parenting (item 2), authoritarian parenting (item 3), over-involvement (item 5, 13), withdrawal (items 6, 7), aversiveness (items 8, 10), psychological control (item 9, 26), autonomy granting (item 12), and warmth (item 14, 16). It also includes the more systemic and general aspect of family cohesion (16, 17, 22), as it is measured in the Family Assessment Device or parental dyad interactions (items 17, 18, 19, 20) [4, 40, 41]. Moreover, it combines some dimensions referring to the family that have been especially salient over the past decades [2, 42, 43]. These constructs include trauma and transgenerational stakes, nefarious secrets (items 24), paradoxical communication (item 11), climate of fear of the outside world (item 23), and the interpersonal role to which the child may be placed (items 15, 27, 28, 29). These dimensions are rarely studied and widely implicated in the psychopathology of adolescents, whatever their symptoms and diagnoses. All these dimensions are distinct from abuse and neglect, already described in other established and validated scales [36, 44]. The items of the ARFIL scale were reviewed by experts in adolescent psychology and psychiatry, pediatrics, and child protection, who gave their opinion on the clinical content, comprehensibility, and clarity. The scale was tested on 25 family situations before implementation in the Family and Care study, and 2 items were added at the end of this pilot test (“Climate of fear, mistrust, hostility of a parent/parents towards the child”, and “The child is reminded that he/she was unwanted or is illegitimate”). The whole scale was resubmitted to the experts, and then translated and back-translated (agreement kappa score = 76.7%).

The clinician scores each of the 30 items categorically (0 = absent, 1 = present), then assesses the overall intensity of at-risk family interactions resulting from the clinical assessment of the patient, scoring from 0 (not present) to 30 (extremely invasive): this is the Intensity score. Then a Diversity score is calculated with the sum of the items present (those that were rated 1).

### ARFIL scale validation

The scale validation methods included the measure of: the latent structure with a principal component analysis; the internal consistency by calculating Cronbach’s alpha; the inter-rater reliability with the intra-class coefficient for each ARFIL score; the concurrent validity (using the Global Assessment of Functioning scale (GAF), a 100-point hypothetical continuum of mental health and functioning included in the DSM); the convergent validity (using the PBI [13]). The ARFIL scale was subjected to a principal component analysis with varimax rotation (Table 1). We retained this orthogonal rotation keeping all ARFIL items for two reasons: one, inspection of the eigenvalues (greater than one) and analysis of the scree plot led us to choose a three-component model bearing, respectively, for 15%, 11.3%, and 9.3% (sum = 35.6%) of total variance, and second, the three main components were highly interpretable clinically.

**Table 1:** Varimax rotated principal component analysis of the ARFIL scale (ARFIL: at-risk family interactions and levers scale)

ARFIL scale component loadings				
ARFIL items (short-cut labels)	Component			Uniqueness
	1	2	3	
1. Educational incoherence	0.511			0.650
2. Difficulties in setting limits/boundaries	0.399			0.803
3. Excessive demands		0.392		0.748
4. Parentification	0.422			0.779
5. Overprotection			0.542	0.594
6. Lack of parental reliability	0.587	0.342		0.535
7. Emotional coldness		0.626		0.583
8. Constant criticism		0.658		0.552
9. Excessive control		0.340	0.589	0.527
10. Inducing guilt		0.615		0.572
11. Paradoxical communication			0.341	0.725
12. Difficulties with separation			0.504	0.730
13. Intrusive proximity-seeking			0.659	0.549
14. Relational instability	0.627			0.534
15. Overly intimate climate			0.344	0.854
16. Abandonment-centered atmosphere	0.505	0.489		0.503
17. Repeated conflicts within the family	0.735			0.422
18. Parenting conflicts regarding the child	0.553		0.352	0.568
19. Loyalty conflicts	0.715			0.402
20. Parents undermining each other	0.741			0.375
21. Parental unpredictability	0.644			0.542
22. Lack of support, comforting, acceptance	0.326	0.580		0.538
23. Climate of fear of the outside world			0.485	0.762
24. Traumatic familial context			0.371	0.815
25. Unwanted or illegitimate child				0.897
26. Excessive fixation on at-risk behaviors		0.463		0.751
27. Re-enactment of familial conflicts			0.515	0.683
28. Unwarranted inspection				0.879
29. Parent(s) threaten(s) with suicide	0.412			0.774
30. Climate of fear toward the child		0.543		0.675

Component 1 = cohesion/conflict; component 2 = love/hostility; component 3 = autonomy/control

### Data analysis

The data analyses were performed using R software (version 4.0.2). Bivariate correlations with Student’s tests were estimated between ARFIL scale Intensity and Diversity scores and the care indicators, as described by the number of hospitalizations and the cumulative duration of those hospitalizations (Table 2). The different diagnoses were described in their prevalence and in bivariate correlation with ARFIL scores, using Student’s tests (Annex 2). Then multivariate regression models were used to estimate effects of ARFIL Intensity and Diversity scores on the number and duration of hospitalizations, with covariates including age, sex, maltreatment (emotional, physical or sexual abuse and neglect), GAF, and psychiatric diagnoses (Table 3). The two psychiatric diagnoses found to have the highest correlations with respective independent variable were schizophrenia/psychotic disorder and pervasive developmental disorder with hospitalization duration. Borderline personality disorder and bulimia nervosa had the highest correlations with hospitalization number. So, they were introduced as co-variables for each correlation, as other diagnoses were grouped and coded as a dummy variable “other diagnoses”. The individual total length of hospitalizations (in days) was estimated using OLS regressions, and the number of hospitalizations using zero-truncated Poisson models on R using GLM and VGLM procedures.

**Table 2:** Correlation between hospitalizations indicators and ARFIL scores/GAF/age (Student tests) and means of hospitalization indicators by maltreatment/sex (Chi-square tests) (ARFIL: at-risk family interactions and levers, GAF: Global Assessment of Functioning)

	Hospitalization number	*p* value	Hospitalization duration	*p* value		Hospitalization number: mean (sd)		*p* value	Hospitalization duration: mean (sd)		*p* value
	Pearson correlation coef		Pearson correlation coef			No	Yes		No	Yes	
ARFIL intensity	0.17	< 0.001	0.24	< 0.001	Emotional abuse	1.3 (0.6)	1.4 (0.8)	0.049	28.3 (21)	36.4 (30.6)	0.002
ARFIL diversity	0.18	< 0.001	0.16	0.001	Physical abuse	1.3 (0.6)	1.5 (0.8)	0.040	29.4 (21.6)	41.8 (37.1)	0.003
ARFIL components					Sexual abuse	1.3 (0.6)	1.5 (0.8)	0.019	29.9 (22.1)	38.3 (35)	0.021
1. Cohesion/conflict	0.11	0.024	0.069	0.158	Neglect	1.3 (0.5)	1.4 (0.7)	0.125	29.1 (19.9)	33.3 (28.3)	0.084
2. Love/hostility	0.13	0.007	0.063	0.195		Men	Women		Men	Women	
3. Autonomy/control	0.071	0.145	0.016	< 0.001	Sex	1.2 (0.6)	1.4 (0.7)	0.043	29.6 (21.9)	33.3 (28)	0.140
GAF	−0.10	0.035	−0.28	< 0.001							
Age	0.01	0.910	−0.05	0.276

**Table 3:** Multivariate modeling of hospitalization number and duration (ARFIL: at-risk family interactions and levers; BN: bulimia nervosa; BPD: borderline personality disorder; PDD: pervasive developmental disorders; SCZ: schizophrenia)

Covariates	Hospitalization duration (OLS regression)						Hospitalization number (zero-truncated Poisson)					
	Model 1		Model 2		Model 3		Model 4		Model 5		Model 6	
	Estimates	*p*	Estimates	*p*	Estimates	*p*	IRR	*p*	IRR	*p*	IRR	*p*
(Intercept)	27.13	0.075	32.14	**0.036**	32.04	**0.033**	0.27	0.294	0.29	0.311	0.38	0.438
Age	− 0.41	0.632	− 0.37	0.663	− 0.29	0.732	1.00	0.961	1.02	0.809	1.02	0.794
Sex (women)	8.57	**0.001**	8.43	**0.001**	8.54	**0.001**	1.07	0.733	1.08	0.732	1.08	0.731
Emotional abuse	2.96	0.254	3.64	0.160	3.62	0.168	1.30	0.157	1.31	0.156	1.31	0.159
Physical abuse	3.69	0.232	4.07	0.194	5.32	0.099	1.13	0.543	1.13	0.544	1.13	0.534
Sexual abuse	5.53	**0.045**	6.17	**0.026**	6.41	**0.020**	1.21	0.297	1.23	0.252	1.23	0.254
Neglect	− 2.50	0.359	− 2.09	0.460	− 0.58	0.845	0.87	0.529	0.82	0.392	0.85	0.488
GAF	− 0.29	**0.040**	− 0.37	**0.008**	− 0.35	**0.012**	0.98	0.106	0.98	0.056	0.98	0.067
SCZ	22.02	** < 0.001**	22.07	** < 0.001**	21.77	** < 0.001**						
PDD	17.24	** < 0.001**	16.99	** < 0.001**	16.51	** < 0.001**						
Diags except SCZ/PDD	0.59	0.912	0.74	0.892	0.86	0.874						
Intensity (ARFIL)	0.42	**0.011**					1.03	**0.040**				
Diversity (ARFIL)			0.29	0.215					1.03	**0.041**		
1. Cohesion/conflict					0.31	0.807					1.12	0.223
2. Love/hostility					− 0.48	0.710					1.10	0.289
3. Autonomy/control					2.42	**0.037**					1.12	0.184
BPD							1.52	**0.030**	1.45	0.056	1.47	0.050
BN							1.91	**0.001**	1.91	**0.001**	1.93	**0.001**
Diags except BPD/BN							1.40	0.629	1.35	0.667	1.39	0.642
Observations	421		421		421		422		422		422	
*R*^2^/*R*^2^ adjusted	0.268/0.249		0.259/0.240		0.265/0.242				NA			
AIC	3837.686		3842.818		3843.557		622.660		622.895		626.980	

Bold = *p* < 0.05

## Results

### Sample characteristics

Most participants were native European adolescents (95%), including 67% (283/425) of young women, with a mean age of 15.5 y.o. (sd = 1.3). Most of them belonged to families with high (201/425, 47.3%) or intermediate (159/425, 37.4%) economic status. The GAF score at entry was 33.1/100 on average (sd = 9.0, [min = 3, max = 55]). This corresponds to major impairment in several areas (e.g., school, judgment, thinking or mood) or to impairment of sense of reality or communication (e.g., speech that is at times illogical, obscure or inappropriate). The abuse scores noted were 46.1% (196/425) for emotional abuse, 21.4% (91/425) for physical abuse, 25.1% (107/425) for sexual abuse, and 70.5% (300/425) for neglect. The patients had been hospitalized on average 1.3 times (sd = 0.68, [1–7]), and the average number of days of one hospitalization was 24.1 days (18.4, [1–206]). The mean number of cumulative hospital days was 31.8 days, (26.2, [1–232]). The patients had received an average of 2.6 diagnoses (1.3, [1–8]). The diagnoses of the sample are presented in Annex 2.

### ARFIL scale validation

The validation steps of the ARFIL scale are described in Annex 3.

For the factorial analysis, the principal component analysis with varimax rotation yielded three main components. Table 1 presents the item loadings on each component. Only loadings ≥ 0.3 were considered significant. Factor 1 includes items related to the overall family group in relation to group cohesion and conflict. The more this dimension is present, the more the family lacks cohesion and is at risk of ruptures: incoherencies, unreliability, unpredictability, abandonment centered atmosphere, conflicts, etc. Factor 2 includes items related to emotional distance with the child. The more this dimension is at risk, the more the child is subjected to affective distancing and hostility, coldness, criticism, control, guilt, fear, lack of acceptance, etc. Factor 3 includes items related to physical and behavioral control of the child and proximity. The more this dimension is increased, the more the parents exert a controlling proximity on the child, overprotection, difficulties with separation, etc.

### Correlations between at-risk family interactions and care indicators

The analysis of the bivariate correlations (Table 2) revealed positive correlations between the ARFIL scores for Intensity and Diversity and the amount of care described by the number of hospitalizations and the number of cumulative days in hospital. This means that high levels of at-risk family interactions are strongly associated with high levels of number and of length of hospitalizations. The factorial components of the ARFIL scale were also correlated with the level of care: the higher the level of family conflict, the greater the number of hospitalizations. The higher the level of hostility toward the child, the higher the number of hospitalizations. The greater the level of control over the child, the longer the hospital stay.

Multivariate models (OLS and zero-truncated Poisson) (Table 3) showed that a high ARFIL Intensity score was associated with a greater number of cumulative hospital days, even after controlling for age, sex, maltreatment, GAF score, and diagnoses (model 1). It was also associated with a greater number of hospitalizations (model 4). The ARFIL Diversity score was positively and significantly associated with the number of hospitalizations (model 5). The medical severity on admission (GAF score) was associated with the length of hospitalization (models 1, 2, 3). Finally, the third component of the ARFIL scale was associated with longer hospital stay (model 3).

## Discussion

### The scale and its components

The ARFIL scale is, to our knowledge, the first assessment tool for psychiatric clinician daily practice about family interactions in such a precise and holistic way at the same time. It provides a clinical-driven grid to help clinician assessing functional and dysfunctional familial climate. This scale combines advantages of self-reports from the child and from parents (such as consideration of feelings and experiences expressed by each member) [13–16] with those from direct observation methods (which ensure better reliability from external observer) [20, 21], while maintaining a high level of feasibility (rating time less than 10 min). Its good psychometric characteristics with strong internal consistency and good inter-rater reliability makes it also a valid and reliable tool. Concurrent validity is evidenced by correlation with the PBI, and convergent validity is supported by high correlations with severity indicators (strongly significant association between ARFIL scale score and GAF scale score). The factorial analysis of the ARFIL scale measured on 425 families revealed 3 factors: the first factor assesses overall family lack of cohesion. The second factor translates the emotional distancing from the child, in the sense of coldness, criticism, lack of acceptance, hostility. The third factor relates to a closer relational distance, physical and behavioral, rather described by the concepts of overprotection, excessive control, difficulties with separation, intrusive proximity.

Exposure to parental conflicts, which is evaluated in the first factor cohesion/conflicts, has a proven impact on the mental health of children and adolescents to the point of being described in the DSM 5 under the name Child Affected by Parental Relationship Distress. In their review, Harold and Sellers [45] studied the impact of these conflicts on the functioning of children. They suggested assessing parental conflict as part of a more general focus on parenting, which the ARFIL scale allows. Moreover, the second and third factors join the historical dimensions of love/hostility and autonomy/control of the works of Schaefer [9] and Baumrind [10]. Although the current study embodied an empirical approach, the factor structure of our scale is also congruent with Schaefer’s circumplex model of parenting, in a way returning to the field’s original roots, and provides a basis for extending it in new research and applications. Furthermore, we can consider a more global reading of these two factors, within the framework of the “stylistic dimension” proposed by Beavers [46]: the love/hostility factor would qualify centrifugal families tending toward hostility and to rejection toward third parties outside the family, while the autonomy/control factor would come under the centripetal style, characterized by the tendency to withdraw within the family and to fight against attempts at separation. Finally, it should be noted that the autonomy/control factor was correlated with the length of hospitalization, which may suggest that hospitalization is experienced as precisely allowing a separation from the family, which is difficult to achieve otherwise. Considering the descriptions of these dimensions, the three factors of the ARFIL scale were called: 1. cohesion/conflicts, 2. love/hostility, 3. autonomy/control.

### Dysparenting and psychiatric care

The Intensity score of the ARFIL scale was correlated with both the number of days in hospital and the number of hospitalizations in both univariate and multivariate analyses. The Diversity score, which is the sum of the observed items, was correlated with length of hospital stay in univariate and multivariate analysis. These findings suggest that dysfunctional family interactions may influence the psychological state of the adolescent, not only as a factor in the onset or severity of the disorder as previously described [2, 4], but also in the extent of subsequent psychiatric care. To date, research into the predictive factors for hospitalization in adolescent psychiatry has focused little on family interactions, or only through the spectrum of maltreatment, or family composition [24–26, 29, 30]. The few studies that have explored the specific links between parenting and hospitalization in psychiatry used the PBI, the Family Assessment Device and the Family Adaptability and Cohesion Evaluation Scale [13, 17, 40]. They suggested, for example, a link between family dysfunction—overprotection and low level of maternal care—and suicidal behavior in psychiatric inpatients [47, 48], or between family cohesion and the risk of hospitalization in patients with schizophrenia or bipolar disorders [31]. In an older work, Hauser et al. [32] described parent–child interactions with less empathy and more devaluing the child in families in which the adolescent was hospitalized in psychiatry, compared with those in which this was not the case. All together, these studies suggest that family interactions have impact on the care process, but usually focus on one family dimension (cohesion or affective support most of time) and did not considered the amount of care needed, while it is considered as an important healthy, politic, and economic challenge [34]. Rice and Tan’s study [33], which explored other family factors favoring the occurrence of hospitalizations (like trauma and family conflicts), suggested that it is important to broaden and deepen the semiology of family dysfunctions that can impact this particular moment of psychiatric care. In revealing a link between ARFIL scores and hospitalization characteristics (length and number), the results of our Family and Care study reinforce the idea that the precise conditions of the family environment will be decisive for a given patient’s care. Studies on the impact of maltreatment and parent–child interactions on child health are often drawn from different fields and remain separated from each other. By distinguishing between risky family interactions measured by ARFIL and maltreatment, our study also brings together distinct but complementary data from these two aspects, to better discern the place of one and the other. More broadly, the multivariate analyses made it possible to measure the specificity and weight of at-risk family interactions alongside other already known risk factors of hospitalization, like maltreatment, medical severity, and psychiatric disorders. A precise description of the family’s symptoms can help identify risk factors before they lead to maltreatment, and thus assist in the prevention and treatment of these adolescents’ disorders.

It is also to be noted that the two Intensity and Diversity scores did not predict the same variables, the second being correlated with the duration of hospital care while the first was correlated with both number and length of hospitalizations. Intensity of at-risk family interactions gives an idea of the overall level of risk as assessed by the clinician, while Diversity assesses the number of interactions that accumulate independently of the intensity of each. This score indicates the number of different directions that therapeutic efforts will need to take, and as such may be more specifically linked to the duration of care during hospitalization, which will aim to address these different points. The score of Intensity would indicate a more general severity, pointing to the amount of care beyond hospitalization.

### Psychiatric diagnoses and at-risk family interactions

Multivariate analyses based on data-driven methods revealed four diagnoses more particularly linked to care indicators. Schizophrenia and pervasive developmental disorders were correlated with length of hospitalization, while bulimia nervosa and BPD were related to number of hospitalizations. This result is entirely consistent with clinical observations, which show that psychotic symptoms take longer to treat in hospital, whereas bulimic or borderline symptoms require both shorter and more repeated hospitalizations. The results of this study provide further evidence that the longer hospital stay for psychotic disorders is not only due to the disease, but also to the environmental determinants themselves. In this, they continue the observations of Tan et al. [31], who revealed the impact of family cohesion in the risk of hospitalizations for schizophrenia, schizoaffective, and bipolar disorders.

Patients with BPD represent a significant proportion of our sample (30%) and our results showed that these patients are involved in frequent hospitalizations. It is usually described that patients with BPD account for 30–50% of all psychiatric inpatients in adolescence [49]. It shares frequent comorbidity and common determinants with bulimia nervosa [50, 51]. Clinically, we observe that these patients often do not tolerate prolonged care, but are frequently re-hospitalized, for example when they attempt suicide again. Moreover, the environment of these patients is known to be marked by discontinuity and chaotic relationships (including premature separations), as well as maltreatment and emotional invalidation [2, 52–54]. In a previous study, we explored the combination of three types of adversity maltreatment, stressful life events (early separation from parents, parental suicide attempt, parental chronic disease), and parental bonding—as predictors of the number of BPD symptoms [2]. Results indicated that cumulative traumatic experiences largely characterize borderline adolescent’s history; and, that all adversity experiences were likely to contribute to BPD symptoms: sexual, physical and emotional abuse, physical and emotional neglect, stressful life events (early separation from parents, parental suicide attempt, parental chronic disease), and parental bonding (low levels of maternal and paternal care, high levels of maternal and paternal control). Among them, the role of emotional abuse, parental suicide attempt, and a decrease in paternal level of care were particularly prominent. The Family and Care study builds on these findings by showing just how important role family relationships play beyond their extreme dysfunction, maltreatment. The multivariate analyses carried out in this study suggested that dysfunctional family interactions contribute to the amount of hospital care, independently of the pathology and its severity. Adverse events that are not maltreatment (such as early separations) or parenting styles play an independent role, which deserves a precise description.

### Limitations and prospects

The study includes limitations, the main one being the lack of other comparators for concurrent validity than the PBI. However, this scale is one of the only self-report scales for these interactions that is validated in the language of the study. In addition, this tool has been largely used in previous studies on parenting in adolescents. Ideally, the use of observational tools such as the Parenting Clinical Observation Schedule would complement this assessment [20]. Moreover, the population studied comes from families of high socio-economic level, and it is necessary to replicate our results in other socio-economic contexts. Socio-environmental determinants impacting psychological health are often attributed to devalued environments, but our study, as well as a previous one on abuse and neglect of adolescents hospitalized in psychiatry [30], shows that maltreatment is also highly represented in families of high-income levels. A recent study even suggests that in more privileged socio-economic environments, the importance of family functioning is even more preponderant alongside genetic factors in the development of psychiatric disorders (in adopted children) [55]. By detailing at-risk family interactions, our scale will enable future studies to measure more precisely the contribution of this socio-economic factor, as much as cultural determinants, which would play their own role and are not explored here [56]. Also, by correlating dysparenting and child health indicators, our study helps to put into perspective the normative aspect of defining parental behaviors as inappropriate. It is part of conceiving them as dysfunctional from the point of view of the child’s health, in the sense of harmonious development, rather than by culturally defined norms. Another limitation of this study is the nature of the sample, which is made up of hospitalized patients. Patients in this sample, with a low GAF score (33.1/100 on average), are representative of typical adolescents hospitalized in a psychiatric department, with high severity and much comorbidity [57, 58]. This makes it a population of choice for describing the diversity of family environments but calls into question the study’s external validity. The applicability of the scale will have to be assessed in other less severe and complex contexts, or in other types of settings like consultations or child protection services (where there is a need to develop measuring tools), including a time of pre-test training. Future studies could also use this scale to attempt to classify patients’ environments according to their diagnoses, with the aim of better understanding their pathways. The literature on this subject is still sparse, even though the family unit is recognized as a necessary focus for clinical attention (which this study emphasizes). If the results of future studies confirm the value of the ARFIL, it could enable a better clinical assessment of a patients’ family environment and of concrete treatment levers that would be in line with the currently developing trend toward global health. It would also enable predictions to be made about the amount of care required in each specific situation, and help clinicians to better identify at-risk family situations, and thus prevent acute decompensation by managing them at an earlier stage of this key period of development. Finally, it could allow to better understand the evaluation of environmental issues in terms of prevention and the cost of foreseeable care from a mental health policy perspective.

### Supplementary Information

Below is the link to the electronic supplementary material.

Supplementary file1 (PDF 236 KB)

## Data Availability

The datasets presented in this study can be found in online repositories. The names of the repository and accession number(s) can be found at: 10.6084/m9.figshare.24968043.v1.

## References

[1] Merikangas KR, He J, Burstein M et al (2010) Lifetime prevalence of mental disorders in U.S. adolescents: results from the national comorbidity survey replication-adolescent supplement (NCS-A). J Am Acad Child Adolesc Psychiatry 49:980–989. 10.1016/j.jaac.2010.05.017 20855043 10.1016/j.jaac.2010.05.017 PMC2946114

[2] Robin M, Douniol M, Pham-Scottez A et al (2021) Specific pathways from adverse experiences to bpd in adolescence: a criteria-based approach of trauma. J Pers Disord 35:94. 10.1521/pedi_2021_35_523 33999657 10.1521/pedi_2021_35_523

[3] Tanaka M, Wekerle C, Schmuck ML, Paglia-Boak A (2011) The linkages among childhood maltreatment, adolescent mental health, and self-compassion in child welfare adolescents. Child Abuse Negl 35:887–898. 10.1016/j.chiabu.2011.07.003 22018519 10.1016/j.chiabu.2011.07.003

[4] Yap MBH, Pilkington PD, Ryan SM, Jorm AF (2014) Parental factors associated with depression and anxiety in young people: a systematic review and meta-analysis. J Affect Disord 156:8–23. 10.1016/j.jad.2013.11.007 24308895 10.1016/j.jad.2013.11.007

[5] Xiao Z, Murat Baldwin M, Wong SC et al (2022) The impact of childhood psychological maltreatment on mental health outcomes in adulthood: a systematic review and meta-analysis. Trauma Violence Abuse. 10.1177/15248380221122816 36123796 10.1177/15248380221122816 PMC10594835

[6] Pinquart M (2017) Associations of parenting dimensions and styles with internalizing symptoms in children and adolescents: a meta-analysis. Marriage Fam Rev 53:613–640. 10.1080/01494929.2016.1247761 10.1080/01494929.2016.1247761

[7] Pinquart M (2017) Associations of parenting dimensions and styles with externalizing problems of children and adolescents: An updated meta-analysis. Dev Psychol 53:873–932. 10.1037/dev0000295 28459276 10.1037/dev0000295

[8] Gilbert R, Widom CS, Browne K et al (2009) Burden and consequences of child maltreatment in high-income countries. The Lancet 373:68–81. 10.1016/S0140-6736(08)61706-7 10.1016/S0140-6736(08)61706-7 19056114

[9] Schaefer ES (1965) A configurational analysis of children’s reports of parent behavior. J Consult Psychol 29:552–557. 10.1037/h0022702 5846126 10.1037/h0022702

[10] Baumrind D (1991) The influence of parenting style on adolescent competence and substance use. J Early Adolescence 11:56–95. 10.1177/0272431691111004 10.1177/0272431691111004

[11] Belsky J, Schlomer GL, Ellis BJ (2012) Beyond cumulative risk: Distinguishing harshness and unpredictability as determinants of parenting and early life history strategy. Dev Psychol 48:662–673. 10.1037/a0024454 21744948 10.1037/a0024454

[12] Robin M, Belbèze J, Pham-Scottez A et al (2021) Paradoxes in borderline emotional dysregulation in adolescence: influence of parenting, stressful life events, and attachment. Front Psychiatry 12:735615. 10.3389/fpsyt.2021.735615 34744826 10.3389/fpsyt.2021.735615 PMC8566741

[13] Parker G, Tupling H, Brown LB (1979) A parental bonding instrument. Br J Med Psychol 52:1–10. 10.1111/j.2044-8341.1979.tb02487.x 10.1111/j.2044-8341.1979.tb02487.x

[14] Buri JR (1991) Parental authority questionnaire. J Pers Assess 57:110–119. 10.1207/s15327752jpa5701_13 16370893 10.1207/s15327752jpa5701_13

[15] Nicolas CC, Jorm AF, Cardamone-Breen MC et al (2020) Parental self-efficacy for reducing the risk of adolescent depression and anxiety: scale development and validation. J Res Adolesc 30:249–265. 10.1111/jora.12521 31246378 10.1111/jora.12521

[16] Parent et al (2017) The Multidimensional Assessment of Parenting Scale (MAPS): Development and Psychometric Properties | SpringerLink. 10.1007/s10826-017-0741-5. Accessed 16 Jun 2022 10.1007/s10826-017-0741-5 PMC5646703 29056840

[17] Olson DH (1985) FACES IV and the circumplex model: validation study. J Marital Family Ther 37:64–80 10.1111/j.1752-0606.2009.00175.x 21198689

[18] Taber SM (2010) The veridicality of children’s reports of parenting: a review of factors contributing to parent–child discrepancies. Clin Psychol Rev 30:999–1010 20655135 10.1016/j.cpr.2010.06.014

[19] De Los RA, Ohannessian CM, Racz SJ (2019) Discrepancies between adolescent and parent reports about family relationships. Child Dev Perspect 13:53–58 10.1111/cdep.12306

[20] Hill C, Maskowitz K, Danis B, Wakschlag L (2008) Validation of a clinically sensitive observational coding system for parenting behaviors: the parenting clinical observation schedule. Parenting 8:153–185. 10.1080/15295190802045469 37124424 10.1080/15295190802045469 PMC10134773

[21] Melby JN, Conger RD (2001) The Iowa Family Interaction Rating Scales: Instrument summary. Family observational coding systems: resources for systemic research. Lawrence Erlbaum Associates Publishers, Mahwah, pp 33–58

[22] Hendriks AM, Van Der Giessen D, Stams GJJM, Overbeek G (2018) The association between parent-reported and observed parenting: a multi-level meta-analysis. Psychol Assess 30:621–633. 10.1037/pas0000500 28627919 10.1037/pas0000500

[23] Zaslow MJ, Weinfield NS, Gallagher M et al (2006) Longitudinal prediction of child outcomes from differing measures of parenting in a low-income sample. Dev Psychol 42:27–37. 10.1037/0012-1649.42.1.27 16420116 10.1037/0012-1649.42.1.27

[24] Borchardt CM, Garfinkel BD (1991) Predictors of length of stay of psychiatric adolescent inpatients. J Am Acad Child Adolesc Psychiatry 30:994–998 1757450 10.1097/00004583-199111000-00019

[25] Reddy R, Ha C, Newlin E, Sharp C (2017) Predictors of length of stay in a psychiatric adolescent treatment program. J Psychiatr Pract 23:342–351 28961663 10.1097/PRA.0000000000000255

[26] Benarous X, Cravero C, Jakubowicz B et al (2020) Durée d’hospitalisation en pédopsychiatrie: étude rétrospective des facteurs prédictifs sur deux ans en unité d’adolescents. Neuropsychiatrie de l’Enfance et de l’Adolescence 68:377–383 10.1016/j.neurenf.2020.03.004

[27] Zeshan M, Waqas A, Naveed S et al (2018) Factors predicting length of stay in an adolescent psychiatric unit, South Bronx, NY: a short report. J Can Acad Child Adolescent Psychiatry 27:142 PMC5896528 29662526

[28] Kylmänen P, Hakko H, Räsänen P, Riala K (2010) Is family size related to adolescence mental hospitalization? Psychiatry Res 177:188–191 20382431 10.1016/j.psychres.2009.01.015

[29] Keeshin BR, Strawn JR, Luebbe AM et al (2014) Hospitalized youth and child abuse: a systematic examination of psychiatric morbidity and clinical severity. Child Abuse Negl 38:76–83 24041456 10.1016/j.chiabu.2013.08.013

[30] Robin M, Bonnardel L, Saintoyant F et al (2022) Facteurs D’adversité Chez des Adolescents Issus de Milieu Aisé Hospitalisés en Psychiatrie. Can J Psychiatry 67:320–322. 10.1177/07067437211064593 10.1177/07067437211064593 PMC9099085 34898281

[31] Tan JHP, Conlon C, Tsamparli A et al (2019) The association between family dysfunction and admission to an acute mental health inpatient unit: a prospective study. Ir J Psychol Med. 10.1017/ipm.2019.41 31511120 10.1017/ipm.2019.41

[32] Hauser ST, Houlihan J, Powers SI et al (1987) Interaction sequences in families of psychiatrically hospitalized and nonpatient adolescents. Psychiatry 50:308–319. 10.1080/00332747.1987.11024363 3423157 10.1080/00332747.1987.11024363

[33] Rice JL, Tan TX (2017) Youth psychiatrically hospitalized for suicidality: changes in familial structure, exposure to familial trauma, family conflict, and parental instability as precipitating factors. Child Youth Serv Rev 73:79–87. 10.1016/j.childyouth.2016.12.006 10.1016/j.childyouth.2016.12.006

[34] Green J, Jacobs B, Beecham J et al (2007) Inpatient treatment in child and adolescent psychiatry—a prospective study of health gain and costs. J Child Psychol Psychiatry 48:1259–1267 18093032 10.1111/j.1469-7610.2007.01802.x

[35] Horton R, Lo S (2015) Planetary health: a new science for exceptional action. The Lancet 386:1921–1922. 10.1016/S0140-6736(15)61038-8 10.1016/S0140-6736(15)61038-8 26188746

[36] Ntinapogias A, Gray J, Durning P, Nikolaidis G (2015) Coordinated Response to Child Abuse & Neglect via Minimum Data Set

[37] American Psychiatric Association D, Association AP (2013) Diagnostic and statistical manual of mental disorders: DSM-5. American psychiatric association, Washington

[38] Pfohl B, Blum N, Zimmerman M (1997) Structured Interview for DSM-IV Personality: SIDP-IV. American Psychiatric Pub

[39] Sheehan DV, Lecrubier Y, Sheehan KH et al (1998) The Mini-International Neuropsychiatric Interview (M.I.N.I.): the development and validation of a structured diagnostic psychiatric interview for DSM-IV and ICD-10. J Clin Psychiatry 59 Suppl 20:22–33 (**quiz 34-57**) 9881538

[40] Epstein NB, Baldwin LM, Bishop DS (1983) The McMaster family assessment device. J Marital Fam Ther 9:171–180 10.1111/j.1752-0606.1983.tb01497.x

[41] Mansfield AK, Keitner GI, Dealy J (2015) The family assessment device: an update. Fam Process 54:82–93. 10.1111/famp.12080 24920469 10.1111/famp.12080

[42] Isobel S, McCloughen A, Goodyear M, Foster K (2021) Intergenerational trauma and its relationship to mental health care: a qualitative inquiry. Community Ment Health J 57:631–643. 10.1007/s10597-020-00698-1 32804293 10.1007/s10597-020-00698-1

[43] Philipp DA, Prime H, Darwiche J (2023) An ultra-brief systemic intervention to address child mental health symptomatology. Fam Process 62:469–482 36959726 10.1111/famp.12875

[44] Bernstein DP, Fink L, Handelsman L et al (1994) Initial reliability and validity of a new retrospective measure of child abuse and neglect. Am J Psychiatry 151:1132–1136. 10.1176/ajp.151.8.1132 8037246 10.1176/ajp.151.8.1132

[45] Harold GT, Sellers R (2018) Annual Research Review: Interparental conflict and youth psychopathology: an evidence review and practice focused update. J Child Psychol Psychiatry 59:374–402. 10.1111/jcpp.12893 29574737 10.1111/jcpp.12893

[46] Beavers R, Hampson RB (2000) The beavers systems model of family functioning. J Fam Ther 22:128–143. 10.1111/1467-6427.00143 10.1111/1467-6427.00143

[47] Freudenstein O, Zohar A, Apter A et al (2011) Parental bonding in severely suicidal adolescent inpatients. Eur Psychiatry 26:504–507. 10.1016/j.eurpsy.2011.01.006 21398097 10.1016/j.eurpsy.2011.01.006

[48] Prinstein MJ, Boergers J, Spirito A et al (2000) Peer functioning, family dysfunction, and psychological symptoms in a risk factor model for adolescent inpatients’ suicidal ideation severity. J Clin Child Psychol 29:392–405. 10.1207/S15374424JCCP2903_10 10969423 10.1207/S15374424JCCP2903_10

[49] Becker DF, Grilo CM, Edell WS, McGlashan TH (2002) Diagnostic efficiency of borderline personality disorder criteria in hospitalized adolescents: comparison with hospitalized adults. AJP 159:2042–2047. 10.1176/appi.ajp.159.12.2042 10.1176/appi.ajp.159.12.2042 12450954

[50] Miller AE, Trolio V, Halicki-Asakawa A, Racine SE (2022) Eating disorders and the nine symptoms of borderline personality disorder: a systematic review and series of meta-analyses. Intl J Eating Disorders 55:993–1011. 10.1002/eat.23731 10.1002/eat.23731 35579043

[51] Spiegel J, Arnold S, Salbach H et al (2022) Emotional abuse interacts with borderline personality in adolescent inpatients with binge-purging eating disorders. Eat Weight Disord 27:131–138. 10.1007/s40519-021-01142-3 33677816 10.1007/s40519-021-01142-3 PMC8860808

[52] Linehan MM (1993) Skills training manual for treating borderline personality disorder. Guilford Press, New York

[53] Porter C, Palmier-Claus J, Branitsky A et al (2020) Childhood adversity and borderline personality disorder: a meta-analysis. Acta Psychiatr Scand 141:6–20. 10.1111/acps.13118 31630389 10.1111/acps.13118

[54] Johnson JG, Cohen P, Chen H et al (2006) Parenting behaviors associated with risk for offspring personality disorder during adulthood. Arch Gen Psychiatry 63:579–587. 10.1001/archpsyc.63.5.579 16651515 10.1001/archpsyc.63.5.579

[55] Myllyaho T, Siira V, Wahlberg K-E et al (2022) Dysfunctional family functioning in high socioeconomic status families as a risk factor for the development of psychiatric disorders in adoptees: the Finnish Adoptive Family Study of Schizophrenia. Soc Psychiatry Psychiatr Epidemiol 57:1367–1377. 10.1007/s00127-020-02016-2 33398497 10.1007/s00127-020-02016-2

[56] Cho Y-B, Haslam N (2010) Suicidal ideation and distress among immigrant adolescents: the role of acculturation, life stress, and social support. J Youth Adolescence 39:370–379. 10.1007/s10964-009-9415-y 10.1007/s10964-009-9415-y 20229228

[57] Tonge BJ, Hughes GC, Pullen JM et al (2008) Comprehensive description of adolescents admitted to a public psychiatric inpatient unit and their families. Aust N Z J Psychiatry 42:627–635. 10.1080/00048670802119770 18612866 10.1080/00048670802119770

[58] Blanz B, Schmidt MH (2000) Practitioner review: preconditions and outcome of inpatient treatment in child and adolescent psychiatry. J Child Psychol Psychiatry Allied Disciplin 41:703–712 10.1111/1469-7610.00658 11039683

