# Supplementary material for: Influence of at-risk family interactions on the course of psychiatric care in adolescence
Source: Eur Child Adolesc Psychiatry. 2024 Feb 2;33(8):2847–57. doi: 10.1007/s00787-023-02330-5 (PMC11272672; doi:10.1007/s00787-023-02330-5)
Supplement: Supplementary file 1 — Supplementary file1 (PDF 236 KB) [file 787_2023_2330_MOESM1_ESM.pdf]

ANNEXES

Annex 1: At-Risk Family Interactions and Levers (ARFIL) scale items and their prevalence (N = 425 families)

AT-RISK FAMILY INTERACTIONS AND LEVERS (ARFIL) SCALE ITEMS (N = 425 families)	N	%	
1 - Educational incoherence	203	47.8	
2 - Important difficulties with setting limits/boundaries or establishing a cohesive educational framework	250	58.9	
3 - Excessive demands (rigid parenting, inappropriate and high standards) or misunderstanding of the child's needs	206	48.5	
4 - Parentification of one or more of the children, role reversal in the parent-child relationship	137	32.2	
5 - Overprotection of the child	119	28.0	
6 - Lack of protection or parental reliability	227	53.4	
7 - The child's emotions are minimized, or there is an absence of emotional sharing and reciprocity; emotional coldness directed at the child	194	45.6	
8 - Constant criticism, disparagement, or pessimism regarding the child	130	30.6	
9 - Family relationships are rooted in excessive control	172	40.5	
10 - Inducing guilt, the child is treated as a scapegoat (ex: the child is blamed for all the problems the family is facing), the parent is presented as good and the child as bad	123	28.9	
11 - Paradoxical communication; words and attitudes contradict each other	235	55.3	
12 - Difficulties with separation	271	63.7	
13 - Constant and intrusive proximity-seeking by a parent	194	45.6	
14 - Relational instability, frequent ruptures between family members	143	33.6	
15 - Overly intimate climate made apparent through seductive attitudes, including of a sexual nature, within the family	72	16.9	
16 - Abandonment-centred familial atmosphere (rejection, abandonment or threats of abandonment, conditional love)	164	38.6	
17 - Repeated conflicts, ongoing or unresolved conflicts within the family involving one of the parents or both	201	47.3	
18 - Parenting conflicts regarding the child	117	27.5	
19 - Loyalty conflicts between the parents themselves or with the grandparents	117	27.5	
20 - Parents undermining each other	134	31.5	
21 - Parental unpredictability	143	33.6	
22 - Lack of support, comforting, acceptance, and listening	155	36.5	
23 - Instilling a climate of fear of the outside world or isolation from it	95	22.3	
24 - Traumatic familial context (including unresolved loss, nefarious secrets)	309	72.7	
25 - The child is reminded that he/she was unwanted or is illegitimate	24	5.6	
26 - Excessive fixation at the idea of the child developing at-risk behaviors	113	26.6	
27 - The parent re-enacts conflicts he/she has with a grandparent or an aunt/uncle in his/her interaction with the child	194	45.6	
28 - Unwarranted inspection of the child for signs of physical or mental health symptoms	40	9.4	
29 - Parent(s) threaten(s) the child with suicide	65	15.3	
30 - Climate of fear, mistrust, hostility of a parent/parents towards the child	86	20.2	
	N	Mean	SD
**A. Score of Intensity (0 - 30)**	425	16.9	8.1
**B. Score of Diversity = sum of the present items (0 – 30)**	425	10.9	5.7

Annex 2: Prevalence of diagnoses and correlation with care indicators, hospitalization number and duration (Pearson correlation coefficients and p-values of Pearson product-moment independence tests).

		Hospitalization number		Hospitalization duration	
**Diagnoses**	**Frequence**	**Pearson corr. Coeff.**	**p-value**	**Pearson corr.**	**p-value**
Major depressive disorder	49.9 %	0.0012	0.981	-0.034	0.487
Anxiety disorders	43.1 %	0.07	0.149	0.06	0.221
Borderline personality disorder	30.1 %	0.19	<0.001	-0.013	0.783
Addictive disorder	29.2 %	0.15	0.001	0.0042	0.932
Pervasive developmental disorder	16.7 %	0.11	0.021	0.3	<0.001
Schizophrenia/psychotic disorder	12.9 %	0.057	0.243	0.34	<0.001
Other personality disorders	12.2 %	-0.054	0.265	-0.047	0.339
Bulimia nervosa	11.3 %	0.19	<0.001	0.065	0.183
Anorexia nervosa	10.1 %	-0.039	0.421	-0.044	0.362
Adjustment disorder	7.1 %	0.053	0.278	0.022	0.653
Post-traumatic stress disorder	6.6 %	0.11	0.03	0.1	0.039
Attention deficit/hyperactivity	4.0 %	0.16	0.001	0.1	0.034
Oppositional defiant disorder	3.5 %	0.037	0.452	0.013	0.793
Gender dysphoria	3.1 %	0.033	0.502	0.056	0.253
Bipolar disorder	2.4 %	-0.00051	0.992	-0.0053	0.913

Annex 3: Scale validation

The internal consistency of the scale was measured using Cronbach's alpha and a reliability coefficient of 0.835 was obtained. To measure interrater reliability, 34 of the 425 patients were seen concurrently by two clinicians who independently scored the ARFIL scale. The intraclass coefficient (ICC) for the intensity score is 0.75 [SD 0.59; 0.85], and the ICC for the diversity score is 0.74 [SD 0.53; 0.86], showing high agreement between the two raters for both scores. The disagreements were discussed clinically and argued to reach a consensus. F-test results indicate no bias for both Intensity ($F = 6.9$, $p < 0.001$) and Diversity ($F = 6.5$, $p < 0.001$) scores. For concurrent validity, we assessed the extent to which the ARFIL scale correlated with the GAF scale (Annex 2). For convergent validity, the PBI was used. It is a self-administered questionnaire widely used to measure the subjective experience of parent-child attachment from the child's perspective [13]. It is a 4-point Likert scale with 25 items on maternal bonding and 25 items on paternal bonding, each with 13 'control' and 12 'care' items (Cronbach alpha coefficient: 0.98). These items are used to generate care and control scores for each parent (ranging from 0 to 39 for control and 0 to 36 for care). Both ARFIL scale scores show significant negative correlations with the GAF. The Intensity score is significantly correlated with the PBI mother's care and mother's control, while the Diversity score is correlated with mother's care, father's care and father's control.

ARFIL	GAF	PBI			
		Mother care	Father care	Mother control	Father control
Intensity	-0.33 ***	-0.4 **	-0.05	0.28 *	0.06
Diversity	-0.22 ***	-0.44 **	-0.28 *	0.24	0.32 *
*p-values : *** <0.001, ** <0.01, * <0.05*					

Convergent and concurrent validity. Correlation coefficients between the ARFIL scale and GAF/PBI scores (ARFIL: At-Risk Family Interactions and Levers; GAF: Global Assessment Functioning, PBI: Parental Bonding Instrument).
